# Electron Screening in Deuteron–Deuteron Reactions on a Zr Target with Oxygen and Carbon Contamination

**DOI:** 10.3390/ma18061331

**Published:** 2025-03-18

**Authors:** Agata Kowalska, Mateusz Kaczmarski, Konrad Czerski, Rakesh Dubey, Gokul Das Haridas, Mathieu Valat, Natalia Targosz-Ślęczka, Paweł Figiel, Justyna Słowik, Jolanta Baranowska

**Affiliations:** 1 Physics Department, Maritime University of Szczecin, Wały Chrobrego 1-2, 70-500 Szczecin, Poland; 2 Institute of Physics, University of Szczecin, Wielkopolska 15, 70-451 Szczecin, Poland; mateusz.kaczmarski@usz.edu.pl (M.K.); konrad.czerski@usz.edu.pl (K.C.); rakesh.dubey@usz.edu.pl (R.D.); gokul.haridas@phd.edu.pl (G.D.H.); mathieu.valat@usz.edu.pl (M.V.); natalia.targosz-sleczka@usz.edu.pl (N.T.-Ś.); 3 Faculty of Mechanical Engineering and Mechatronics, West Pomeranian University of Technology, Piastów 19, 70-310 Szczecin, Poland; pawel.figiel@zut.edu.pl (P.F.); justyna.slowik@zut.edu.pl (J.S.); jolanta.baranowska@zut.edu.pl (J.B.)

**Keywords:** electron screening, DD fusion, crystal lattice impurities, XRD

## Abstract

The electron screening effect is responsible for a significant increase in the nuclear reaction rates in metals at very low energies. This is dependent on the local crystal structure of the metallic target and the occurrence of defects or additional elemental impurities in the crystal. Here, we studied the deuteron–deuteron fusion reactions on zirconium targets previously implanted with carbon and oxygen ions. The ^2^H(d,p)^3^H reaction yield was measured at two deuteron energies, 8 and 20 keV, in order to determine the strength of the electron screening effect and its dependence on the density of the implanted impurities. We found that carbon implantation strongly reduced the experimentally determined screening energy, while oxygen implantation had the opposite effect. These results are especially important for the application of nuclear fusion in metallic environments at very low energies.

## 1. Introduction

The cross sections of charged-particle-induced nuclear reactions at low energies are determined by the probability of penetration through the Coulomb barrier. This results in an abrupt exponential decrease in the size of the cross section, thereby lowering the projectile energies, which can be diminished at sufficiently low energies due to the electron screening effect caused by the presence of the surrounding electrons. The screening of the charges of reacting nuclei by the electrons in a specific environment, enhances the measured cross section compared to the equivalents for bare nuclei. This effect was initially discussed in an analysis of the dense astrophysical plasmas in the interior of stars [1], in which the reaction rates can be increased by many orders of magnitude [2]. A similar effect has been observed in laboratory studies of various nuclear reactions, originally performed on gaseous targets [3], where only bound electrons can contribute to the screening effect. In contrast, experiments carried out using metallic targets and quasi-free conduction electrons have shown greater reductions in the Coulomb barrier, described according to the experimentally determined screening energy [4,5,6,7]. The reported values were strongly dependent on the target material but did not correlate with physical properties of the material such as its electrical conduction, density, or diffusion coefficient [8]. Only self-consistent dielectric function theory [9] that consistently considers the polarization of bound and conduction electrons and the screening resulting from the lattice atoms can describe this phenomenon correctly [10]. However, the absolute values of the screening energy have been systematically underestimated by a factor of about three compared with the experimental data [7,10]. This discrepancy was clearly demonstrated in results obtained on the ^2^H(d,p)^3^H reaction in deuterated Zr, which is very stable and homogeneous and can easily be produced through deuterium implantation to make ZrD_2_ stoichiometric. The experimental screening energy varied between 210 and 350 eV [9,11,12], whereas the theoretical prediction was about 110 eV [10].

The situation changed with the use of an ultra-high vacuum technique for accelerator experiments [13]. During precise experimentation, the Zr target surface was kept atomically clean and additionally sputtered with an argon beam. The resulting screening energy of about 105 +/− 15 eV agreed with the theoretical prediction. On the other hand, it has also been shown that the screening energy can increase to as much as 400 eV in long-term irradiation experiments. This result was associated with crystal lattice defects induced by the deuteron beam and small amounts of carbon or oxygen impurities. This is likely to have led to an increase in the effective electron mass due to localization of the nearby electrons, changing the band structure of the metallic target. The observed gain in the screening energy was explained through an increase in the effective electron mass, which was a result of the collective interaction between many electrons in the metallic target.

Independently, similar lattice-dependent effects have been found in other nuclear reactions using inverse reaction kinematics [14], in which annealed and cold-rolled palladium foils were used as the targets. A high screening energy was determined only in the case of a defected (cold-rolled) target, while no effect was found in non-defected (annealed) foil. The conclusion was that cold rolling creates a large number of voids, dislocations, and grain boundary defects in which deuterium atoms can be trapped.

Apart from the presence of defects, the type of crystal structure also seems to play an important role in sub-barrier nuclear reactions. For example, it was observed that a hexagonal structure of titanium resulted in a higher screening energy than that in a Ti cubic structure with deuterium located at the octahedral sites [15]. Other works in which other fcc deuterated metals i.e., Pd and Ni, were used also reported increased cross sections [4,5,10,13].

In our previous work [16], the changes in the crystal lattice and the defect depth profiles of Zr targets after deuteron, carbon, and oxygen implantation were systematically studied by means of positron annihilation spectroscopy (PAS) and X-ray diffraction (XRD). We demonstrated that the deuteron beam creates a uniform layer of crystal lattice vacancies down the beam range. Additional implantation of carbon ions did not change the density depth profile of the vacancies, indicating that saturation was achieved as a result of a balance between the repair and formation of new defects. In the case of oxygen implantation, the saturation level of the vacancies was slightly reduced, and the target depth distribution significantly exceeded the deuteron beam range, which allowed us to conclude that the vacancy–oxygen clusters migrated to deeper target layers. The XRD patterns matched both hexagonal close-packed (hcp) α–Zr and face-centered cubic (fcc) δ–Zr, represented by ZrH_1.5–1.66_ stoichiometry, with the deuterons occupying tetrahedral positions. Grazing-angle XRD additionally revealed that deuteron implantation caused the transfer of oxygen atoms to deeper target layers, in agreement with the PAS observations.

The main goal of the present study is to continue our previous experiments and explain the influence of oxygen and carbon impurities on the electron screening effect. Special attention has been paid to carefully measuring the nuclear reaction rates for DD fusion after additional carbon and oxygen implantation. The results indicate that apart from dislocations and vacancy-type defects, the occurrence of impurities within the crystal structure may also modify the screening energy strongly, with an increase after oxygen implantation and a decrease after implanting carbon. This study is complemented by relevant structural data obtained through an XRD analysis.

## 2. Materials and Methods

### 2.1. The Experimental Setup

The experiments were performed at the eLBRUS Experimental Physics Center of the University of Szczecin using a linear accelerator equipped with an electron-cyclotron resonance ion source (ECRIS), which delivers high-current light ion beams with a long-term energy resolution of several eV. The system operates in ultra-high vacuum (UHV) conditions, provided by a differential pumping system producing a vacuum in the order of 10^−10^ mbar. Using additional cooling methods, e.g., a titanium sublimation pump, LN_2_-cooled elements, and an ion pump, the system is able to reach the 10^−11^ mbar vacuum. During the experiment, the target chamber pressure was 8 × 10^−9^ mbar without the beam and 6 × 10^−8^ mbar during irradiation.

Two deuteron beams with energies of 20 keV and 8 keV and currents on the Zr target of 40 µA and 80 µA, respectively, were extracted, focused, and separated using a 90° analyzing magnet. The ion energy was defined by the voltage at the ion source. Due to the reduction in the reaction cross section by more than 2 orders of magnitude at a lower projectile energy, we increased the beam current for a lower deuteron energy. The analyzing magnet was an electromagnet; therefore, the current used was proportional to the B field in the magnet. The ion beam was shaped and focused using a series of steerers, apertures, and lenses on the target covering a 0.25 cm^2^ spot. The 1 mm thick Zr target was positioned using a four-axis manipulator at an angle of 30° to the beam and parallel to the charged particle silicon detector.

**Figure 1:**
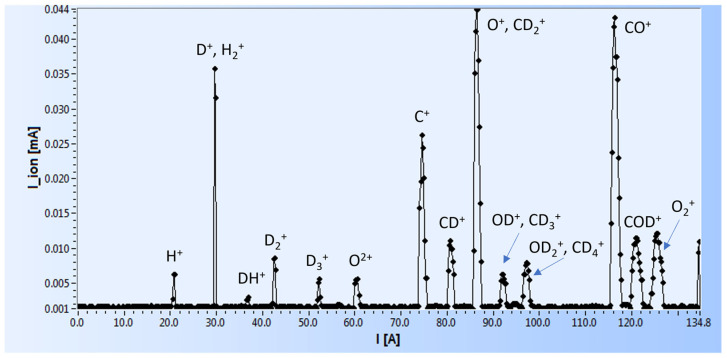
Magnetic mass spectrum of CO_2_ gas ionized in the ECRIS source at 20 kV obtained using the analyzing magnet (on the x-axis, the electromagnet current is used, which is proportional to the B-field).

**Figure 7:**
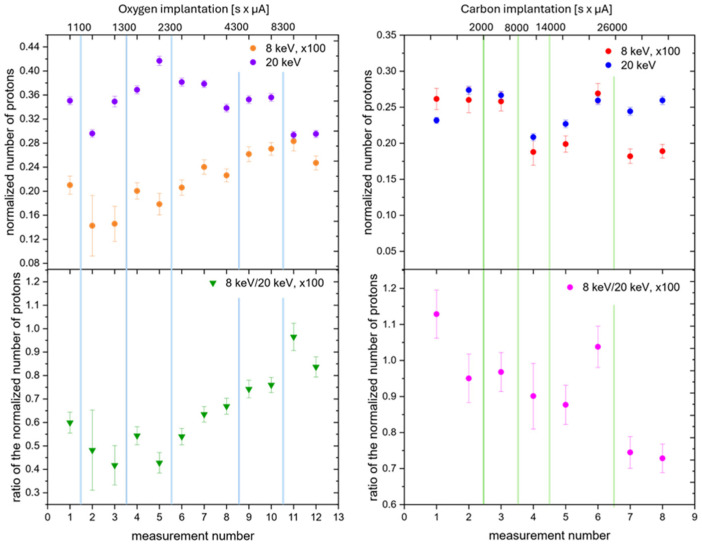
Measurements of enhancement factors resulting from the electron screening effect defined as the ratio of the number of protons detected in the DD reaction at 8 keV and 20 keV normalized to the incident deuteron number. The experiment was performed on a deuterated Zr target implanted stepwise with O (**left part**) and C (**right part**) ions.

The zirconium target was irradiated with deuterons until the saturation level for stoichiometry in the ZrD_2_ was reached. Next, it was implanted with 20 keV of atomic carbon and oxygen gradually, up to 6.5 × 10^17^ atoms per cm^2^ for carbon and 2 × 10^17^ atoms per cm_2_ for oxygen. To produce the carbon and oxygen beams, CO_2_ gas was used. The magnetic mass spectrum of the ionized gas obtained by the analyzing magnet is presented in Figure 1. Due to them having the same mass, the oxygen line contained an additional contribution from CD_2_ of less than 5%. To measure the progress of carbon and oxygen implantation into the Zr target, a special dose unit, the product of the beam current and the irradiation time [µA·s], was applied (see Figure 7). This unit could be then converted into an absolute number of atoms by multiplying it by a factor of 2.5 × 10^13^ at/cm^2^, which resulted from the fluence of the ion beam corresponding to the 1 µA current of single ionized atoms.

**Figure 2:**
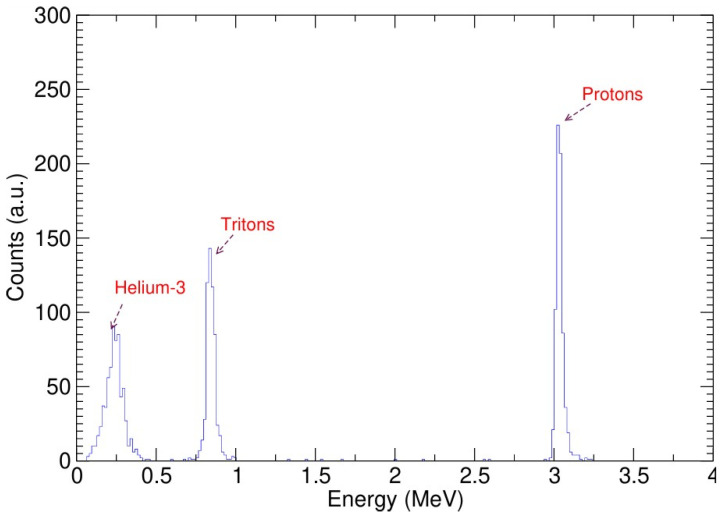
Energy spectrum measured using 100 µm thick Si detector and 1.2 µm thick Al absorption foil under 8 keV D2 beam.

After each implantation step, measurements of the thick target yields of the ^2^H(d,p)^3^H reaction at deuteron energies of 20 and 8 keV were performed to determine the corresponding screening energy. An EG ORTEC silicon detector, with a thickness of 100 μm and a detection area of 100 mm^2^, situated at a backward angle of 135°, was employed to measure all of the charged particles emitted: the protons, tritons, and ^3^He particles produced by the DD fusion reactions. The analog NIM bin system was used to process the energy signal generated in the detector, and data were acquired via the TUKAN MCA. The experimental energy spectrum measured at a deuteron energy of 8 keV obtained using the Si detector with a 1.2 μm thick Al absorption foil in front is shown in Figure 2. The aluminum foil was placed in front of the detector in order to absorb the backscattered deuterons and prevent the detector from being destroyed. A comprehensive Geant4 Monte Carlo simulation [17] and SRIM/TRIM [18] calculations were also performed to determine the depth distribution of the implanted deuterons, as well as the carbon and oxygen atoms.

### 2.2. Determination of the Screening Energy Using the Two-Point Method

**Figure 3:**
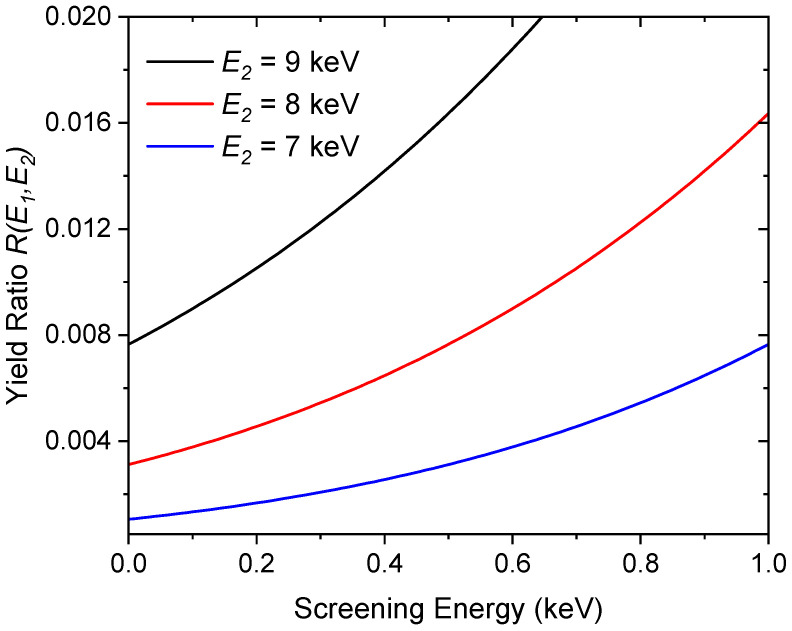
Normalized yield ratio according to Equation (5) calculated for different deuteron energies.

The screening energy was evaluated after each implantation step using the so-called two-point method. For this purpose, 20 keV and 8 keV deuteron beams were chosen. At the higher deuteron energy, the electron screening effect can be neglected. Therefore, the measured reaction yield can be used for the determination of the deuteron density in the target. At the lower deuteron energy, the electron screening contribution reaches significant values; thus, a comparison of the count rates between these two different beam energies allows us to calculate the screening energy (see Figure 3).

The thick target yield $Y\left(E\right)$ measured directly in the experiment is an integral over the reaction cross section σ(E) and the range of projectiles in the target R:

(1) $$Y\left(E\right)=n\int_{0}^{R}\sigma (E)dx=n\int_{E}^{0}\frac{\sigma (E)}{dE/dx}dE=n\int_{0}^{E}\frac{\sigma (E)}{\left|dE/dx\right|}dE=n\int_{0}^{E}\frac{\sigma (E)}{C\sqrt{E}}dE$$

Here, n is the deuteron density in the target, and the expression $\left|dE/dx\right|=C\sqrt{E}$ denotes the stopping power function, which is proportional to the square root of the projectile energy in the studied energy region [7,18]. Under electron screening, the cross section will be enhanced, and the screened yield reads as follows:

(2) $$Y_{scr}\left(E\right)=n\int_{0}^{E}\frac{\sigma_{scr}(E)}{C\sqrt{E}}dE=\frac{n}{C}\int_{0}^{E}\;\frac{\frac{1}{\sqrt{\left(E+U_{e}\right)\;E}}\;S\left(E\right)\;exp\left(-\sqrt{\frac{E_{G}}{E+U_{e}}}\right)}{\sqrt{E}}dE$$

where transformation of the cross section into the astrophysical S-factor is applied, considering a Gamow energy $E_{G}$ equal to 986 keV for the deuteron–deuteron collision system. This transformation separates the tunneling probability through the Coulomb barrier and wavelength dependences from the nuclear effects included in the S-factor. Since the S-factor can be considered constant and the screening energy $U_{e}$ is much smaller than the projectile energy E, we obtain

(3) $$Y_{scr}\left(E\right)=\frac{n\;S(E)}{C}\int_{0}^{E}\;\frac{exp\left(-\sqrt{\frac{E_{G}}{E+U_{e}}}\right)}{E^{3/2}}dE$$

which can be calculated analytically:

(4) $$Y_{scr}\left(E\right)=\frac{2n\;S(E)}{C\sqrt{E_{G}}}\left[\exp\left(-\sqrt{\frac{E_{G}}{E+U_{e}}}\right)-exp\left(-\sqrt{\frac{E_{G}}{U_{e}}}\right)\right]≃\frac{2n\;S(E)}{C\sqrt{E_{G}}}\exp\left(-\sqrt{\frac{E_{G}}{E+U_{e}}}\right)$$

Here, the second term in square brackets can be neglected if $U_{e}\ll E$. As the astrophysical S-factor is known, the formula above can be used to fit the experimental thick target yield and determine the screening energy $U_{e}$. According to Equation (4), measurement of the thick target yield at two different low-energy points will be enough to estimate $U_{e}$. Additionally, calculating their ratio will make the result independent of the stopping power factor C and the deuteron density:

(5) $$R\left(E_{1},E_{2}\right)=\frac{Y_{scr}\left(E_{1}\right)}{Y_{scr}\left(E_{2}\right)}≃\frac{S(E_{1})}{S(E_{2})}\exp\left(\sqrt{\frac{E_{G}}{E_{2}+U_{e}}}-\sqrt{\frac{E_{G}}{E_{1}+U_{e}}}\right)$$

The expression above can be further simplified if one of the measurements is performed at a higher projectile energy, for which the screening effect can be neglected, and the S-factor does not change strongly. In the case of deuteron–deuteron reactions, a deuteron beam energy of 20 keV can be used for monitoring measurements, at which the electron screening effect can be neglected.

In Figure 3, the yield ratio $R\left(E_{1},E_{2}\right)$ is presented for different $E_{2}$ energies ($E_{1}=20\;keV$) depending on the screening energy in the ^2^H(d,p)^3^H reaction. Considering the strongly reduced reaction cross section at reduced deuteron energies and increased measurement times, the best choice for practical applications, allowing for the fast determination of $U_{e}$, is $E_{1}=20\;keV,\;E_{2}=8\;keV$.

## 3. Results

### 3.1. Geant4 vs. SRIM/TRIM Calculations

A custom-tailored physics list was used for the Geant4 simulations. G4EmStandardPhysics_Option4 was used as the electromagnetic physics list, as it offered detailed treatment of the stopping powers, range calculations, and secondary particle production. This model involves detailed treatment of the secondary particles produced, as the stochastic nature of these secondaries can introduce additional variability into the ion’s trajectory and energy deposition. The ion beams were simulated using the General Particle Source (GPS) module in Geant4. The beam profiles were modeled as having an elliptical cross section with dimensions of 5 mm × 7 mm. The ions were singly ionized and characterized by a Gaussian energy distribution with a central energy and a standard deviation of 5% from the mean value.

TRIM calculations were performed to find the implantation profile of the particle in the target. A ZrD_2_ target with a density of 5.72 g/cm^3^ was used in both the Geant4 and TRIM simulations. The stopping power values were taken from SRIM2013.00. A total of 10,000 ions were fired. Monoenergetic ions were used.

**Figure 4:**
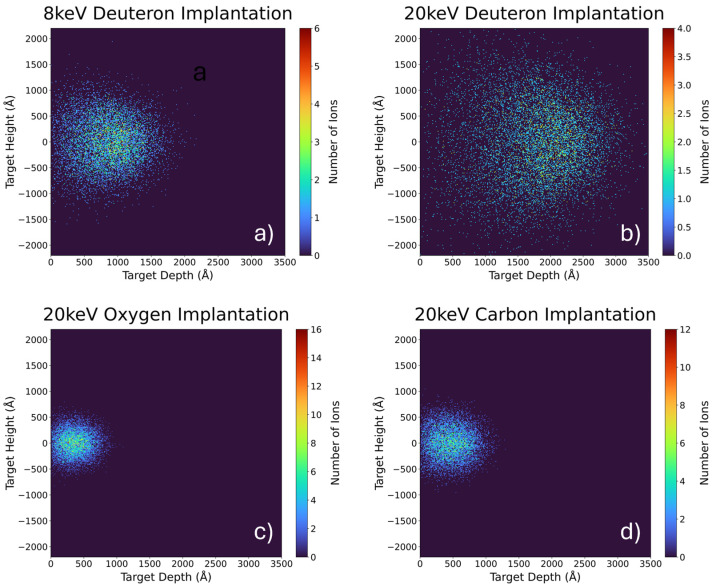
Two-dimensional profiles of the ion implantation of the ZrD_2_ target obtained using SRIM/TRIM (**a**) after 8 keV deuteron implantation; (**b**) after 20 keV deuteron implantation; (**c**) after 20 keV oxygen implantation; and (**d**) after 20 keV carbon implantation.

In Figure 4, the 2D profiles obtained using SRIM/TRIM (version 2013.00) are presented.

**Figure 5:**
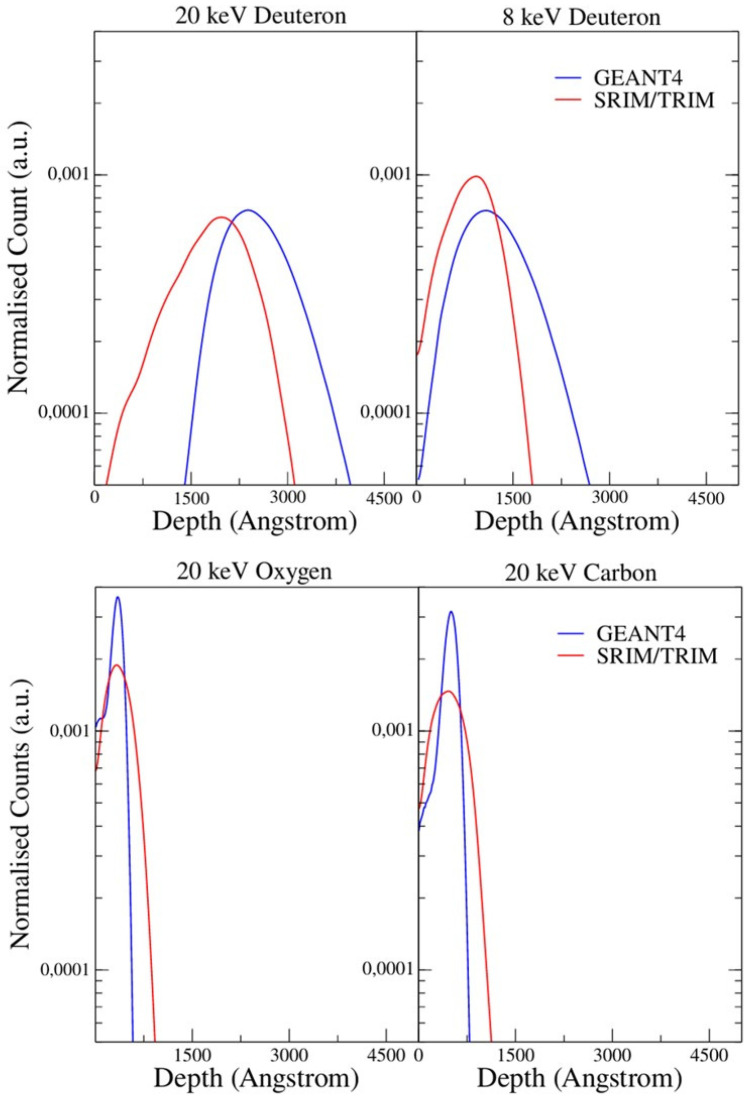
Comparison of 1D depth distributions calculated using Geant4 and SRIM/TRIM. The beam parameters are the same as in Figure 4.

A comparison of the 1D depth distributions obtained using Geant4_11.3.0 and TRIM/SRIM (version 2013.00) is presented in Figure 5. The range of implanted ions using both toolkits is given in Table 1. The discrepancy between the ranges obtained using both simulations is most significant for deuterons, and it increases with a decreasing deuteron energy. It arises from the way that Geant4 handles low energies, in which the careful choice of the step size plays a significant role. When using too small a step size, the energy loss process loses its stochastic nature, and the statistical variation in the range is underestimated. On the other hand, larger step sizes (~10% of the total range) cause the calculation of the energy loss to be averaged over larger distances, and the exact position of the scattering events within the steps becomes less precise. This leads to the formation of discrete pattern formations in the simulation results, which we have called “chunking”. As the total range of ions decreases, establishing a balance in the step size for accurately modeling the proper physics process becomes increasingly difficult. In this case, the ion ranges obtained for low energies using SRIM/TRIM, which uses the experimental values, seem to be more accurate.

Despite the significant differences between the depth profiles of the implanted ions calculated using the two different methods, in both cases, the deuteron ranges are much larger than those for the implanted carbon and oxygen atoms (see Table 1). Therefore, the DD fusion yields should be sensitive to the changes in the electron band structure induced by implanted impurities.

### 3.2. XRD Analysis

**Figure 6:**
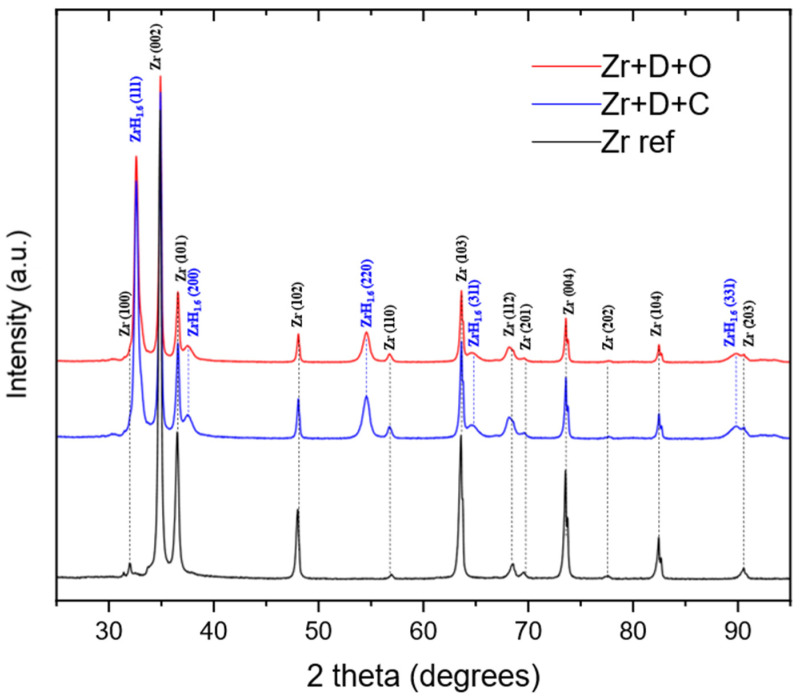
XRD patterns of the reference Zr target (black line), the Zr target implanted with deuteron and carbon (blue line), and the Zr target implanted with deuteron and oxygen.

The reference Zr target, as well as the targets that underwent deuteron and oxygen implantation and deuteron plus carbon implantation, was examined using X-ray diffraction (XRD). The Bragg–Brentano geometry was applied within a 2Θ angle range of 25–95°. CuKα radiation was used. The obtained data were handled using the X’Pert HighScore (v. 2.2.1.) software provided by Panalytical [19]. In Figure 6, a comparison between the non-irradiated (reference) and both irradiated targets is presented.

Similarly to our previous study [16], the preferred orientation of the reference target was (002). The detected sequence of XRD patterns in the Zr target corresponds to a hexagonal close-packed (hcp) crystal structure. The detected plane indices corresponding to the hcp crystal structure were (100),(002), (101), (102), (110), (103), (112), (201), etc. The positions of the hcp Zr patterns were similarly shifted for the targets implanted with carbon and oxygen atoms, resulting in the difference in the lattice’s parameter values (see Table 2) calculated according to [20]. In both cases, irradiation led to the expansion of the c parameter and slight contraction of the a parameter. For the deuteron-implanted Zr target, no significant changes in the crystal lattice constants could be observed, which was in contradiction with the DFT calculations, which predicted the anisotropic expansion of a and the contraction of c [21]. The additional planes that appeared in the XRD patterns of the irradiated targets were characteristic of a face-centered cubic structure (fcc) and corresponded to δ–phase zirconium deuteride (denoted as ZrH_1.5_-ZrH_1.66_ stoichiometry), in which the tetrahedral positions are occupied by deuterium. Unfortunately, the XRD patterns of the implanted Zr targets, collected in the Bragg–Brentano geometry, did not contain significant evidence of zirconium oxide or carbide formation. This may primarily have been due to the significantly lower implantation depth of these ions compared to that of deuterons (see Table 1), the much deeper penetration of the X-rays, and the amount by which these phases were below the detection threshold of the XRD technique. The uncertainty in the lattice parameters of about 0.002 Å was assessed according to a small change in the position of one of the XRD lines fitting within its uncertainty.

### 3.3. Electron Screening Energies

Figure 7 shows the proton counting rates in the ^2^H(d,p)^3^H reaction performed on a deuterated Zr target gradually implanted with oxygen and carbon atoms, normalized with respect to the deuteron target current and measured for deuteron energies of 20 and 8 keV. They are proportional to the thick target yields defined by Equations (1) and (2). The uncertainties in the experimental data are much larger than statistical errors, which results from the differences in the deuteron densities at different beam spots after changes in the beam energies. In the lower part of Figure 7, the corresponding 8 keV/20 keV yield ratios are also displayed. Whereas the ratio systematically increases with the implantation dose of oxygen (left part), indicating an increase in the screening energy, for carbon implantation, we observe the opposite tendency, corresponding to a reduction in the screening energy. According to Equation (5) (see Figure 3), the changes in the screening values can be estimated as follows: for oxygen, U_e_ = 300 eV ↗ 600 eV, and for carbon, U_e_ = 600 eV ↘ 400 eV. Surprisingly, the screening energy alterations arise for very low implantation fluences, reaching the cumulative values 2 × 10^17^ at/cm^2^ for oxygen and 6.5 × 10^17^ at/cm^2^ for carbon atoms. These amounts were not detectable in the XRD patterns but were enough to observe changes in the crystal lattice constants (Table 2).

## 4. Discussion and Conclusions

In the present work, the influence of small carbon and oxygen impurities on the enhancement in the nuclear reaction yield at very low projectile energies due to the electron screening effect was studied. We compared the thick target yields of the ^2^H(d,p)^3^H reaction determined on a deuterated Zr target additionally implanted with oxygen and carbon atoms. This study is a continuation of our previous paper [16], which showed that deuterium implantation in Zr results in uniformly distributed crystal lattice vacancies that may be responsible for the enhanced electron screening observed in nuclear reactions in metallic environments. Hereby, the role of oxygen and carbon impurities, which can additionally stabilize the crystal vacancies, was not clear.

In the present work, the energies of the implanted ions are much lower since the electron screening effect increases the reaction cross sections at reduced projectile energies. Consequently, the top target layers mostly contribute to the measured thick target yields and are sensitive to changes in the screening energy.

Our measurements show that even a small amount of implanted oxygen atoms—corresponding to 1/4 of the total implanted dose—already leads to an increase in the screening energy, ultimately reaching a very high value of about 600 eV, which is six times higher than the theoretical and experimental estimations obtained for a defect-free Zr target [13]. In the case of carbon implantation, the observed tendency was the opposite—the screening energy decreased down to about 400 eV. These data are burdened with relatively large experimental uncertainties, arising from the non-uniformities of the Zr targets and the time limitations on the measurements, which only allowed for rapid determination of the screening energy. We applied a method based on only two deuteron energy points in representing the enhancement in the nuclear cross section due to the electron screening effect.

The number of implanted oxygen and carbon atoms was so small that we could not recognize any XRD patterns related to oxides or carbides. However, the lattice constants were significantly changed compared to those of the virgin and deuterated samples. Calculations of the depth distributions of the implanted ions in Zr performed using Geant4 and SRIM/TRIM codes showed large differences, which were especially visible for the deuteron beam. Probably, this was due to the different stopping power values applied.

Nevertheless, the trends in the experimentally determined screening energies are very significant and demonstrate a method through which we can modify nuclear reaction cross sections at very low energies. This is of significant importance for commercial applications of nuclear fusion. As presented in Figure 3, the reaction yields at deuteron energies of several keV can be increased even more than twofold at higher screening energy values. At lower deuteron energies of several eV, the deuteron–deuteron fusion rates can then be enhanced, even by more than 20 orders of magnitude [22], leading to the possible technical utilization of deuteron–deuteron fusion at lower temperatures. On the other hand, this enhancement may be very unstable due to the easy change in the local chemical stoichiometry of the materials used and the enhanced diffusion of impurities [23].

The observed enhancement in the nuclear reaction yield is assigned to the trapping of deuterium in the vacancies and the localization of electrons in the crystal lattice defects due to local changes in the electron band structure of metals. This can be understood according to the larger curvature of the electron valence band and consequently an increase in the effective electron mass, leading to the higher electron screening energy and the significant enhancement in the reaction yield. This effect can additionally be influenced by oxygen and carbon impurities, which form stable vacancy–deuterium–impurity systems [24]. According to the Thomas–Fermi model [25], the screening energy is proportional to the square root of the effective electron mass. This is just a phenomenological explanation of the effects observed. For a more sophisticated approach, microscopic DFT calculations would certainly be needed, but this is beyond the scope of the present work.

## Figures and Tables

**Table 1:** Mean range calculated using SRIM/TRIM and Geant4.

Implanted Ion	Mean Range in SRIM [Å]	Mean Range in Geant4 [Å]
Deuteron at 20 keV	1800	2500
Deuteron at 8 keV	860	1250
Oxygen at 20 keV	370	290
Carbon at 20 keV	470	446

**Table 2:** Lattice parameters of hcp Zr before and after implantation. The uncertainty in the lattice constants amounts to about 0.002 Å.

Target	a [Å]	c [Å]
Zr (reference)	3.208	5.217
Zr implanted with D	3.209	5.243
Zr implanted with D and O	3.195	5.302
Zr implanted with D and C	3.195	5.294

## Data Availability

The original contributions presented in this study are included in the article. Further inquiries can be directed to the corresponding author.
